# Short-term outcomes and safety of radiotherapy for immediate breast reconstruction with autologous flap transfer following breast-conserving surgery

**DOI:** 10.1186/s12885-021-07915-4

**Published:** 2021-03-02

**Authors:** Shu-Ling Zhang, Jun Song, Yan-Ru Wang, Yi-Jia Guo, Jian-Zhu Zhao, Li Sun, Le-Tian Huang, Jie-Tao Ma, Cheng-Bo Han

**Affiliations:** Department of Oncology, Shengjing Hospital of China Medical University, Shenyang, 110004 China

**Keywords:** Breast cancer, Immediate breast reconstruction, Autologous flap, Postoperative radiotherapy

## Abstract

**Background:**

The outcomes of immediate autologous breast reconstruction (IABR) after partial mastectomy followed by postoperative radiotherapy (RT) in terms of aesthetics, treatment-related complications, and local control are unclear. In this study, we evaluated the efficacy of IABR after partial mastectomy with or without breast RT, and thus the impact of radiation on autologous flap transfer.

**Method:**

A retrospective cohort study involving consecutive breast cancer patients who underwent IABR after partial mastectomy between July 2011 and December 2017 at Shengjing Hospital was performed. Patients were divided into two groups based on whether or not they received RT after IABR. We compared aesthetic outcomes and changes in the flap size over the three-dimensional coordinates at various timepoints (pre-RT, 1, 6, and 12 months post-RT), as well as postoperative complications, survival, and recurrence rates between the two groups.

**Results:**

In total, 84 breast cancer patients were enrolled, with 32 patients in the RT group and 52 in the non-RT group. At a median follow-up time of 33.3 months, no significant difference was found in the rate of regional recurrence between the two groups (3.13% vs. 3.85%, *P* = 1.00), and no local recurrences occurred in either group. At the timepoints pre-RT, 1, and 6 months post-RT (approximately 4, 7, and 12 months after IABR, respectively), 77 (91.7%), 70 (83.3%), and 83 (98.8%) patients, respectively, had achieved very good or good cosmetic outcomes, and only changes in breast skin color at 1 month after RT significantly differed between the RT and non-RT groups, with very good or good cosmetic result rates of 62.5% vs. 96.2%, respectively (*P* < 0.001). No significant difference in the reduction of flap size was observed at any timepoint between the two groups. There were no significant differences between the two groups in the rates of postoperative complications including necrosis of the flap, infection, hematoma, or seroma (all *P* > 0.05). Additionally, no grade 3 or greater RT-associated adverse events occurred during or after RT.

**Conclusion:**

RT following IABR provides aesthetically satisfactory results without intolerable adverse complications and may safely be performed in patients who underwent IABR after partial mastectomy.

**Supplementary Information:**

The online version contains supplementary material available at 10.1186/s12885-021-07915-4.

## Background

Breast-conserving surgery (BCS) combined with postoperative radiotherapy (RT) has been commonly used in patients with early-stage breast cancer [1, 2]. BCS is sometimes called lumpectomy, quadrantectomy, partial mastectomy, or segmental mastectomy depending on how much tissue is removed. Some selective breast cancer patients with larger tumor-to-breast ratios can undergo oncoplastic surgery after partial mastectomy, thus avoiding total mastectomy and obtaining better breast appearance [3–5]. Relational data have shown that the rate of positive margins in patients undergoing oncoplastic BCS was significantly lower than that of traditional BCS, and there were no differences in either overall survival rates or relapse-free survival rates between the two techniques [4]. Tissue-expander/implant and autologous tissue reconstruction are the two major surgical approaches to breast reconstruction. Previous studies have demonstrated that patients who received autologous tissue grafting are more satisfied with their breasts and attain higher levels of mental and sexual health than those who received implant-based reconstruction [6–8].

Various studies [9–13] have shown that immediate autologous breast reconstruction (IABR), including autologous fat grafting and myocutaneous flap transfer, is an alternative method for some patients who received BCS, with better aesthetic results and safety, lower rates of surgical complications, and no significant differences in survival compared with traditional BCS or implant-based reconstruction. Additionally, IABR has other advantages over delayed reconstruction such as a shorter total operative time, less scarring, and decreased psychological distress for the patients [14, 15].

However, the current indications for adjuvant RT after oncoplastic BCS still refer to the criteria for traditional BCS [16]. Importantly, it is unclear whether adjuvant RT after IABR following BCS would have harmful effects on autologous tissue flaps, improve survival rates, or reduce local regional recurrence in patients who have undergone reconstructive surgery. Recently, nearly 20% of breast conserving patients in our institution have undergone IABR. It is necessary to assess the effects of adjuvant therapies, especially RT, on the outcomes of autologous tissue reconstruction. Thus, the aim of this study was to observe the efficacy, aesthetic effect, changes in flap size, and complications of RT after IABR following BCS.

## Methods

### Study population selection

**Fig. 1:**
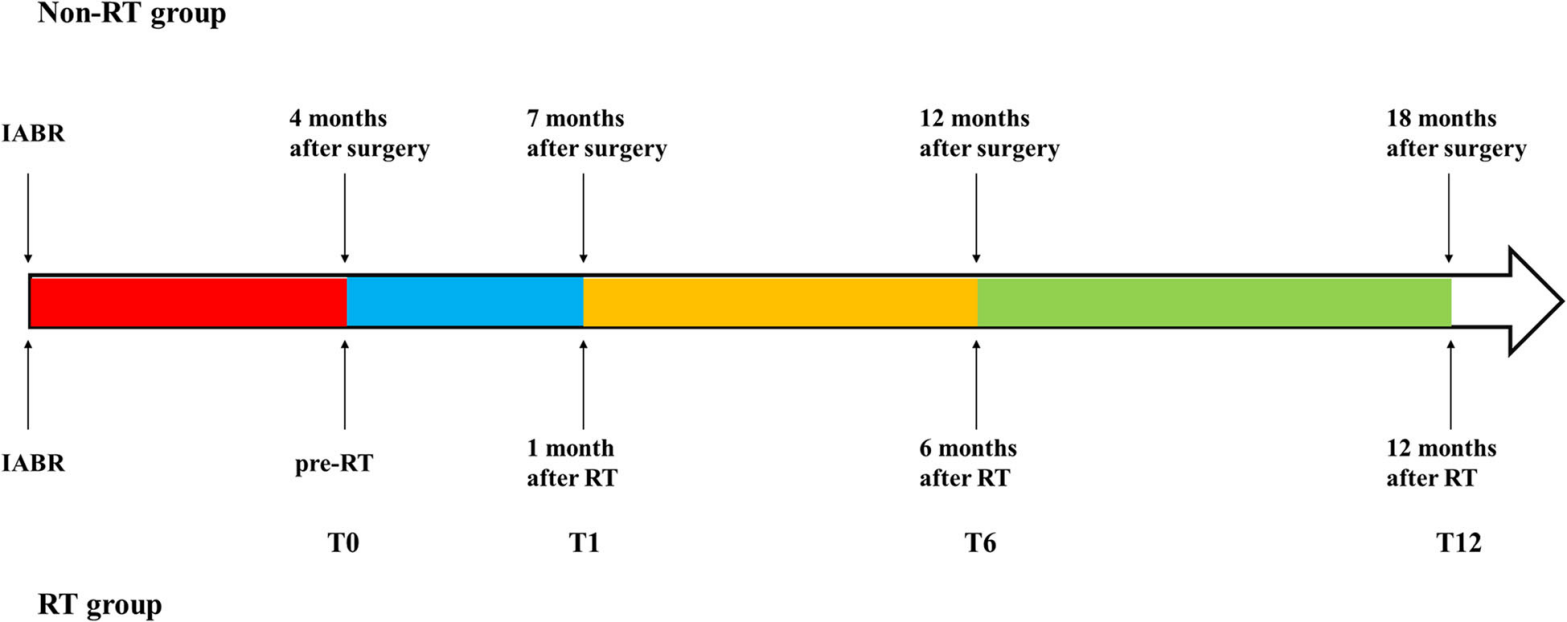
The relative timepoints for the RT and non-RT groups. T0, pre-RT (4 months after surgery); T1, 1 month post-RT (7 months after surgery); T6, 6 months post-RT (12 months after surgery); T12, 12 months post-RT (18 months after surgery). RT, radiotherapy; IABR, immediate autologous breast reconstruction

We retrospectively reviewed clinical data of breast cancer patients who underwent partial mastectomy and IABR at Shengjing Hospital of China Medical University between July 2011 and December 2017. All enrolled patients underwent a wide tumor excision with negative margins and immediate reconstruction with a latissimus dorsi (LD) flap or free dermal fat graft (FDFG). All postoperative patients received primary systematic therapy including adjuvant chemotherapy, targeted therapy, and endocrine therapy, according to their clinicopathological staging and molecular classification. Breast RT or not depended on the indication of RT after BCS and the surgical margin. In the early days of IABR at our center, surgeons generally allowed patients with small-sized breasts and wide tumor excision (safety margin > 2 cm) and lack of other RT indications to avoid RT, because the original breast tissue of such patients is almost completely resected after partial mastectomy. Patients were divided into two subgroups, the RT and non-RT groups, according to whether or not RT was given. Patients in the RT group received whole breast RT at approximately 4 months after surgery, with or without boost irradiation to the tumor bed. The radiation dose was scheduled as 50 Gy in 2-Gy per fraction for the whole breast RT phase, and 10 Gy in 2-Gy per fraction for the boost phase. All eligible patients underwent chest three-dimensional computed tomography (CT) scans at four timepoints: pre-RT (T0), 1 month post-RT (T1), 6 months post-RT (T6), and 12 months post-RT (T12) (corresponding to 4, 7, 12, and 18 months post-surgery, respectively), and the timepoint of IABR was defined T − 4 (Fig. 1). All patients were followed up for more than 2 years after IABR. The study protocol was approved by the relevant institutional review committees, and all participants provided signed comprehensive informed consent forms to participate in the retrospective cohort study.

### Data collection and main outcome measures

The data collected included age, performance status, tumor stage, histological and molecular classification, methods of breast reconstruction, extent of resection, adjuvant chemotherapy, adjuvant RT and RT doses, dates and sites of recurrences or metastases, date and cause of death, aesthetic outcomes, safety, and date of last follow-up.

The main outcome measures were aesthetic outcomes, the reduction in flap size compared with the initial implanted flap, and complications of surgery and/or RT. The cosmetic results after IABR were evaluated by patients using the four-point professional aesthetic assessment scale [17, 18] at the timepoints T0, T1, and T6. Specifically, each category (scar, shape, nipple position, symmetry, and skin color) was classified as good or very good, satisfactory, or poor. An informal questionnaire was used to grade the patient’s level of satisfaction with the aesthetic results. A scale was used wherein the overall result was defined and rated from 0 to 3 (0 = very good, 1 = good, 2 = satisfactory, and 3 = poor). The overall result of each patient was the mean of the category scores. We assessed flap size by calculating the 3D coordinates of the flaps in the CT scanning image, where the X, Y, and Z axis arrows were the left, anterior, and cephalad directions, representing the diameters of length, width, and thickness, respectively. The centers of the 3D coordinates were chosen on the maximum diameter of length plane at the axial position. Then the maximum diameters of length, width and thickness for the flap were measured independently by one radiologist and one radiation oncologist based on three-dimensional CT scans performed at T − 4, T0, T1, T6, and T12. The reduction in maximum length (LR_i_) was calculated by subtracting the maximum diameter of length for the flap at T − 4 (L_f_) from maximum diameter of length at the different timepoints (L_i_), i.e., LR_i_ = L_i_ − L_f_. The reduction in maximum width (WR_i_ = W_i_ − W_f_) and the reduction in maximum thickness (TR_i_ = T_i_ − T_f_) were calculated similarly.

### Statistical analysis

All statistical analyses were performed using the SPSS statistical software version 25.0 (IBM Corp., Armonk, NY, USA). Continuous variables are expressed as the mean ± standard deviation (SD) and were analyzed using Student’s t-test. Survival curves of disease progression were drawn using the Kaplan–Meier method and compared using the log-rank test. The chi-square test and Fisher exact test were used for inter- and intra-group analyses, respectively. The accepted level of significance was *P* < 0.05.

## Results

### Patient characteristics

In total, 84 patients with stage I–III breast cancer who underwent BCS and IABR were included. Their mean age was 46 (range: 28–72) years. Among them, 57 patients underwent IABR with LD flaps and 27 underwent IABR with FDFG. There were 32 and 52 patients in the RT and non-RT groups, respectively. Among the 52 cases in the non-RT groups, three patients with N2 or T3 disease did not receive RT due to their personal choice. All patients in the RT group received whole breast RT at the median time of 4.1 (range: 1.0–7.5) months after IABR, among which 12 patients received boost irradiation to the tumor bed following whole breast RT. Clinical characteristics of patients in the two groups are summarized in Table 1. There were no significant differences between the two groups in terms of age, tumor size, stage, or the expression of progesterone receptor (PR), estrogen receptor (ER), and Her-2.

**Table 1:** Clinicopathological characteristics of patients in the RT group and non-RT group

Characteristic	RT group (*n* = 32)	Non-RT group (*n* = 52)	χ2	*P* value
Age (years)			0.17	0.897
≥ 45	18	30		
< 45	14	22		
Pathological stage			1.622	0.480
I	13	28		
II	15	40		
III	4	4		
T stage			2.278	0.334
pT1	24	32		
pT2	7	18		
pT3	1	1		
N stage			4.045	0.123
pN0	18	40		
pN1	10	9		
pN2	4	3		
ER			0.526	0.468
Positive	21	38		
Negative	11	14		
PR			0.526	0.468
Positive	21	38		
Negative	11	14		
Her-2			0.348	0.555
Positive	6	14		
Negative	25	38		

*RT* radiotherapy

### Aesthetic outcomes

The aesthetic outcomes for all patients are shown in Table S1 in the Supplementary Appendix. At T0, T1, and T6, the overall cosmetic results were considered to be very good or good in 91.7, 83.3, and 98.8% of cases, respectively, satisfactory in 7.1, 15.5, and 1.2% of cases, respectively, and poor in 1.2, 1.2, and 0% of cases, respectively. No significant difference in cosmetic effect was found between the RT and non-RT groups at the three timepoints, except for the breast skin color change at 1 month post-RT (Table 2). A lower rate of very good or good cosmetic results of skin color presented in the RT group compared with the non-RT group, but the difference had disappeared at T6.

**Table 2:** Cosmetic results of the RT group and non-RT group

Cosmetic results, n (%)	pre-RT		*P* value	1 month after RT		*P* value	6 months after RT		*P* value
	RT group	Non-RT group		RT group	Non-RT group		RT group	Non-RT group	
Scar			0.138			0.103			0.057
Very good	10 (31.2)	9 (17.3)		12 (37.5)	11 (21.2)		21 (65.6)	23 (44.2)	
Good	22 (68.8)	43 (82.7)		20 (62.5)	41 (78.8)		11 (33.4)	29 (55.8)	
Satisfactory	0	0		0	0		0	0	
Poor	0	0		0	0		0	0	
Shape			0.209			0.192			0.198
Very good	10 (31.2)	10 (19.2)		13 (40.6)	14 (26.9)		20 (62.5)	25 (48.5)	
Good	22 (68.8)	42 (80.8)		19 (59.4)	38 (73.1)		12 (37.5)	27 (51.5)	
Satisfactory	0	0		0	0		0	0	
Poor	0	0		0	0		0	0	
Symmetry			0.356			0.094			0.239
Very good	11 (34.4)	13 (25.0)		15 (46.9)	15 (28.8)		19 (59.4)	24 (46.2)	
Good	21 (65.6)	39 (75.0)		17 (53.1)	37 (71.2)		13 (40.6)	28 (53.8)	
Satisfactory	0			0	0		0	0	
Poor	0			0	0		0	0	
Skin color			0.761			< 0.001			0.185
Very good	8 (25.0)	10 (19.2)		1 (3.1)	15 (28.8)		23 (71.9)	32 (61.5)	
Good	23 (71.9)	41 (78.8)		19 (59.4)	35 (67.3)		8 (25.0)	20 (38.5)	
Satisfactory	1 (3.1)	1 (2.0)		12 (37.8)	2 (3.9)		1 (3.1)	0	
Poor	0	0		0	0		0	0	
Nipple position			0.454			0.082			0.075
Very good	8 (25.0)	10 (19.2)		12 (37.5)	15 (28.8)		20 (62.5)	23 (44.2)	
Good	22 (68.8)	41 (78.8)		18 (56.3)	37 (71.2)		11 (34.4)	29 (55.8)	
Satisfactory	1 (3.1)	1 (2.0)		2 (6.2)	0		1 (3.1)	0	
Poor	1 (3.1)	0		0	0		0	0	
Overall			0.441			< 0.001			0.424
Very good	6 (18.8)	7 (13.5)		0	11 (21.1)		7 (21.9)	15 (28.8)	
Good	22 (68.8)	42 (80.8)		20 (62.5)	39 (75.0)		24 (75.0)	37 (71.2)	
Satisfactory	3 (9.3)	3 (5.7)		11 (34.4)	2 (3.9)		1 (3.1)	0	
Poor	1 (3.1)	0		1 (3.1)	0		0	0	

*RT* radiotherapy

### Flap size changes

**Fig. 2:**
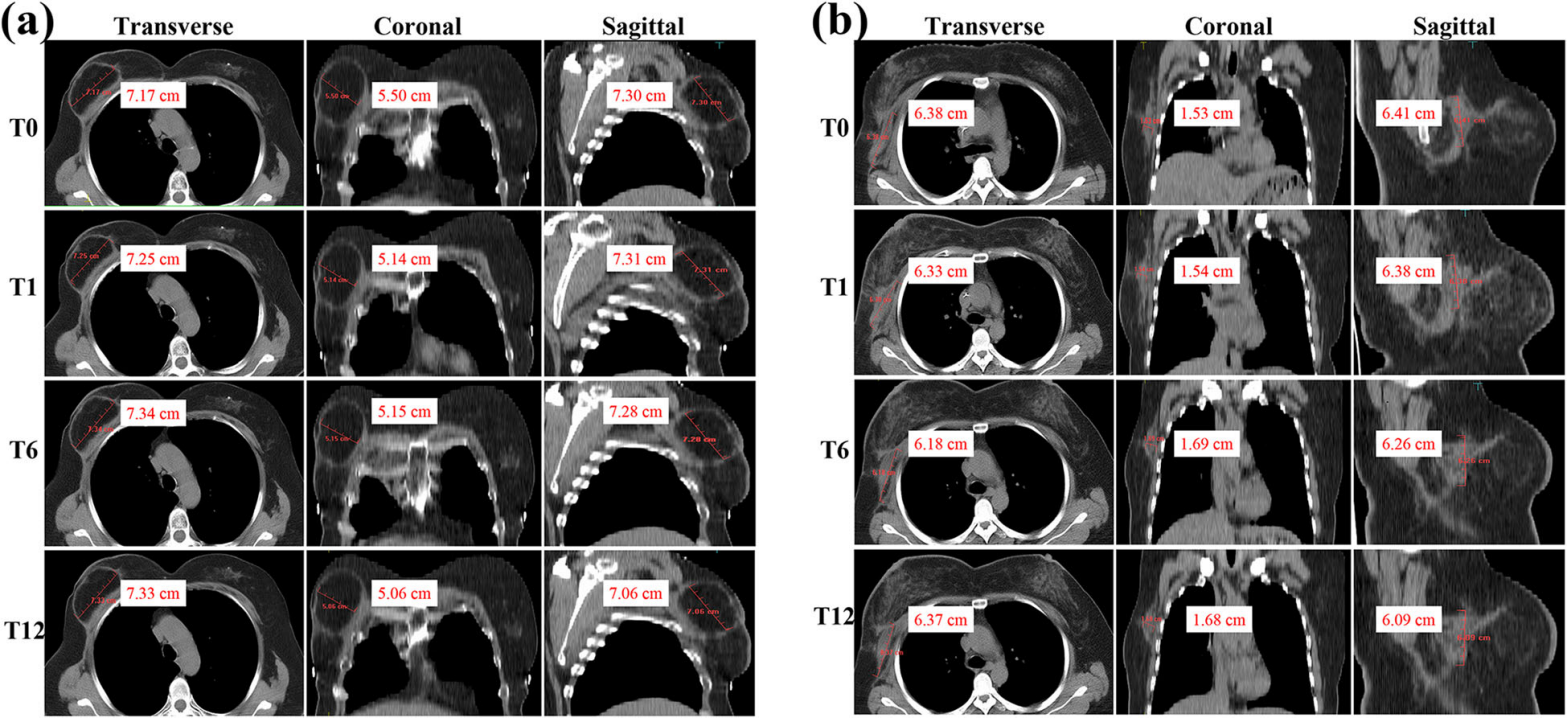
Breast CT scans of two patients who underwent IABR with FDFG (**a**) and LD flap (**b**), respectively. The maximum length (left), maximum width (center) and maximum thickness (right) of the flap were measured based on three-dimensional CT scans at T0 (pre-RT), T1 (1 month post-RT), T6 (6 months post-RT), and T12 (12 months post-RT). CT, computed tomography; IABR, immediate autologous breast reconstruction; FDFG, free dermal fat graft; LD, latissimus dorsi

**Fig. 3:**
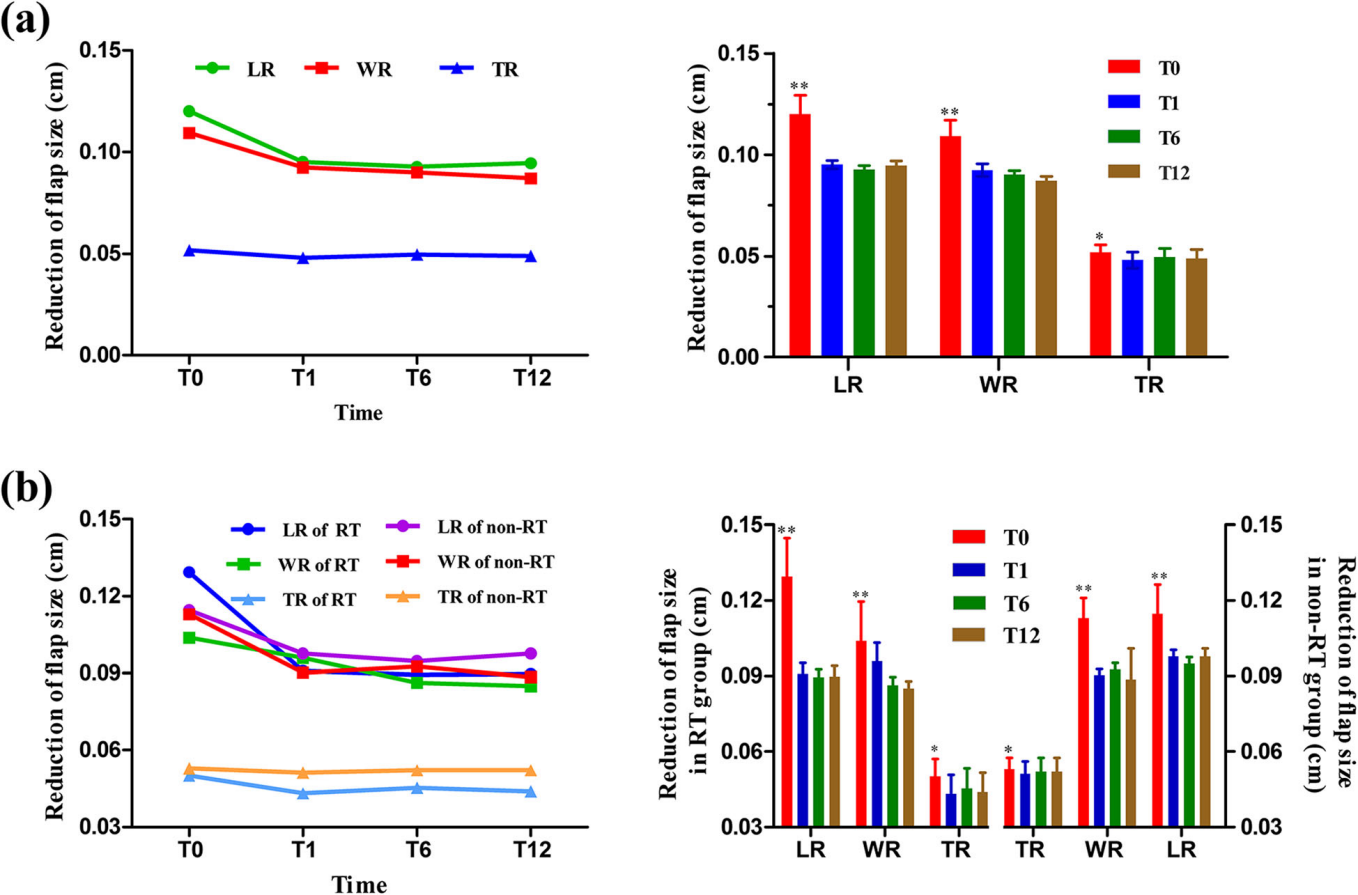
The reduction of flap size along the maximum length, width, and thickness at T0 (pre-RT), T1 (1 month post-RT), T6 (6 months post-RT), and T12 (12 months post-RT) for all patients (**a**), and for patients in the two groups (**b**). *P < 0.05; **P < 0.01. RT, radiotherapy; LR, reduction in maximum length; WR, reduction in maximum width; TR, reduction in maximum thickness

The data of flap size reduction after surgery in the three-dimensions of the maximum length, width, and thickness for the whole group are summarized in Table 3. Figure 2 shows breast CT scans of two patients at pre-RT and post-RT time points. There were no significant differences in the reduction in any of the three dimensions at T1, T6, or T12 (*P* > 0.05). However, there were significant differences in each of these indices at all three timepoints when compared to T0 (all *P* < 0.05, Fig. 3a).

**Table 3:** Changes in flap size among all patients

LR	Mean ± SD (cm)	*P* value	WR	Mean ± SD (cm)	*P* value	TR	Mean ± SD (cm)	*P* value
LR_0_	0.1202 ± 0.08437	0.004	WR_0_	0.1094 ± 0.07072	0.016	TR_0_	0.0518 ± 0.03527	< 0.001
LR_1_	0.0951 ± 0.01942		WR_1_	0.0924 ± 0.02886		TR_1_	0.0481 ± 0.03759	
LR_0_	0.1202 ± 0.08437	0.002	WR_0_	0.1094 ± 0.07072	0.006	TR_0_	0.0518 ± 0.03527	0.036
LR_6_	0.0927 ± 0.01839		WR_6_	0.0901 ± 0.01846		TR_6_	0.0495 ± 0.04077	
LR_0_	0.1202 ± 0.08437	0.004	WR_0_	0.1094 ± 0.07072	0.002	TR_0_	0.0518 ± 0.03527	0.017
LR_12_	0.0946 ± 0.02284		WR_12_	0.0871 ± 0.01905		TR_12_	0.0489 ± 0.04033	
LR_1_	0.0951 ± 0.01942	> 0.05	WR_1_	0.0924 ± 0.02886	> 0.05	TR_1_	0.0481 ± 0.03759	> 0.05
LR_6_	0.0927 ± 0.01839		WR_6_	0.0901 ± 0.01846		TR_6_	0.0495 ± 0.04077	
LR_12_	0.0946 ± 0.02284		WR_12_	0.0871 ± 0.01905		TR_12_	0.0489 ± 0.04033	

*SD* standard deviation, *LR* reduction in maximum length, *WR* reduction in maximum width, *TR* reduction in maximum thickness, *LR*_*0,1,6,12*_ LR of the flap at the timepoints pre-radiotherapy (RT); 1 month after RT, 6 months after RT, and 12 months after RT, respectively, *WR*_*0,1,6,12*_ WR of the flap at the timepoints pre-RT, 1 month after RT, 6 months after RT, and 12 months after RT, respectively, *TR*_*0,1,6,12*_ TR of the flap at the timepoints pre-RT, 1 month after RT, 6 months after RT, and 12 months after RT

The data of flap size reduction in the RT and non-RT groups are summarized in Table 4. No significant difference in the reduction of flap size was found between the two groups at any timepoint (*P* > 0.05). Furthermore, no significant difference in the reduction of flap size was found at T1, T6, or T12 within each group. However, in both groups, the reduction of flap size along the maximum length, width, and thickness at T1, T6, and T12 were significantly different from those at T0 (*P* < 0.05, Fig. 3b).

**Table 4:** Changes in flap size within the two groups

Items	Flap size changes, Mean ± SD (cm)		*P* value
	RT group (*n =* 32)	Non-RT group (*n =* 52)	
LR_0_	0.1294 ± 0.08658	0.1146 ± 0.08332	0.440
LR_1_	0.0909 ± 0.02022	0.0977 ± 0.01864	0.122
LR_6_	0.0894 ± 0.01777	0.0948 ± 0.01863	0.190
LR_12_	0.0897 ± 0.02321	0.0977 ± 0.02228	0.119
WR_0_	0.1038 ± 0.08809	0.1129 ± 0.05822	0.568
WR_1_	0.0959 ± 0.04110	0.0902 ± 0.01777	0.379
WR_6_	0.0863 ± 0.01827	0.0925 ± 0.01835	0.133
WR_12_	0.0850 ± 0.01566	0.0885 ± 0.02090	0.422
TR_0_	0.0500 ± 0.03910	0.0529 ± 0.03304	0.718
TR_1_	0.0431 ± 0.04269	0.0512 ± 0.03417	0.371
TR_6_	0.0453 ± 0.04458	0.0521 ± 0.03847	0.461
TR_12_	0.0438 ± 0.04346	0.0521 ± 0.03837	0.359

*RT* radiotherapy, *SD* standard deviation, *LR*_*0,1,6,12*_ reduction in the maximum length of the flap at the time points pre-RT, 1 month after RT, 6 months after RT, and 12 months after RT, respectively, *WR*_*0,1,6,12*_ reduction in the maximum width of the flap at the time points pre-RT, 1 month after RT, 6 months after RT, and 12 months after RT, respectively, *TR*_*0,1,6,12*_ reduction in the maximum thickness of the flap at the time points pre-RT, 1 month after RT, 6 months after RT, and 12 months after RT

### Complications of IABR and safety of RT

The incidence rates of postoperative complications from IABR were 1.2% for partial necrosis of the flap, 1.2% for infection, 3.6% for hematoma, and 1.2% for seroma. There were no significant differences in the overall rate of complications (9.4% vs. 5.8%; relative risk [RR]: 1.345, 95% confidence interval [CI]: 0.574–3.148; *P* = 0.670) or in the rate of each complication between the RT and non-RT groups (Table 5).

**Table 5:** Postoperative complications in the RT and non-RT groups

Items	Complications, n (%)		*P* value
	RT group (*n* = 32)	Non-RT group (*n* = 52)	
Partial necrosis	1 (3.1)	0 (0)	1.00
Infection	0 (0)	1 (1.9)	0.62
Hematoma	1 (3.1)	2 (3.8)	1.00
Seroma	1 (3.1)	2 (3.8)	0.38
Total	3 (9.4)	3 (5.8)	0.67

*RT* radiotherapy

No grade 3 or greater RT-related adverse events (AEs) occurred during or after RT. The most common AEs were grade 2 or less radiation pneumonitis (25%), radiation dermatitis (37.5%), and leukopenia (3.1%). Five (15.6%) and three (9.4%) cases experienced grade 1 and 2 radiation pneumonitis, respectively, within 3 months after RT; 12 (37.5%) patients who received a tumor bed boost experienced grade 1 radiation dermatitis during RT. Grade 1 leukopenia occurred in only one patient during the first week of RT.

### Survival and recurrence

**Fig. 4:**
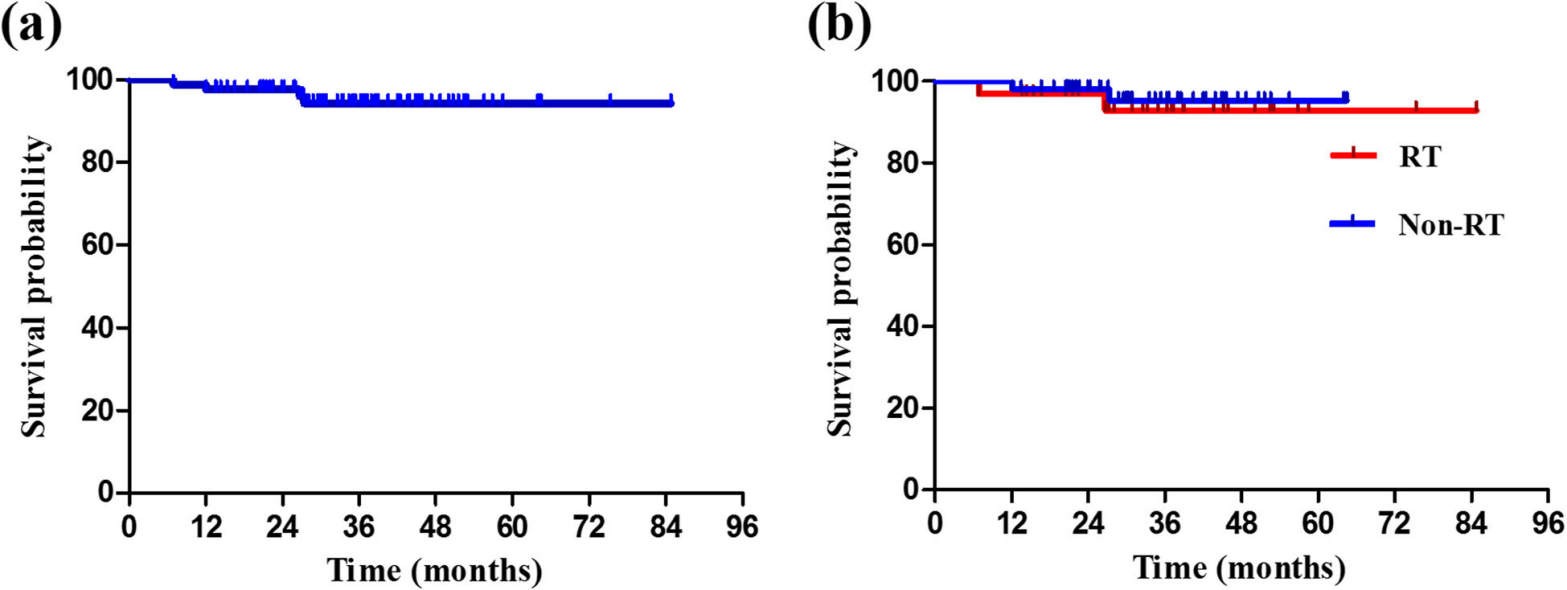
Kaplan–Meier curve of disease-free survival for all patients (**a**), and for RT group versus non-RT group (**b**). RT, radiotherapy

At a median follow-up time of 33.3 months (95% CI: 29.1–37.5), all 84 cases were alive, and the median disease-free survival (DFS) had not been reached (Fig. 4). The 2-year DFS rates were 97.6, 96.9, and 98.1% for the overall cohort, the RT group, and the non-RT group, respectively. Four cases experienced distant or regional metastases, but no patients developed local recurrence. In the RT group, one patient experienced distant metastases and another experienced regional recurrence at 6.9 and 26.7 months after surgery, respectively. In the non-RT group, two cases experienced regional recurrence at 12.1 and 27.4 months after surgery, respectively. No significant difference was found in the regional recurrence rate between the RT and non-RT groups (3.13% vs. 3.85%; RR: 0.871, 95% CI: 0.172–4.419; *P* = 1.00).

## Discussion

IABR plays a vital role for breast cancer patients in terms of preserving anatomical landmarks, minimizing scar fibrosis, reducing the total number of operations, and improving patient satisfaction and psychological outcomes. This is primarily because the long-term success and aesthetic satisfaction of autologous reconstruction are superior to delayed or alloplastic reconstruction [19–21]. However, there are scant data on the effect of RT on autologous flaps. In this cohort study, we focused on assessing the efficacy and safety of IABR with or without RT. The results showed that subsequent RT was well tolerated by patients who received IABR after BCS, and that patients were satisfied with the aesthetic outcomes, suggesting that RT had no serious effects on autologous tissue flaps.

Recently, autologous grafting has become much more widely used in breast cancer patients who need breast reconstruction because of its improved oncological outcomes and reconstruction quality [22, 23]. Additionally, surgeons are increasingly inclined to perform immediate breast reconstruction for patients who need adjuvant RT [24]. Studies have shown significantly improved aesthetic outcomes of grafted autologous flaps after mastectomy [25, 26]. However, aesthetic outcomes of RT following IABR have been contradictory [27]. Several previous studies [28–30], which included a total of 96 patients, evaluated aesthetic outcomes of reconstructed breasts after mastectomy by quartile scores based on patients’ self-evaluation; roughly 77.1% of the cases reported very good or good outcomes after RT. In our study, the rate of aesthetic outcomes being considered very good or good was the lowest for the RT group (62.5%) at 1 month after RT (T1) but reached 96.9% at 6 months after RT (T6), which was higher than previously reports. The differences might be caused by different radiation technique, methods of breast tumor resection, and/or breast reconstruction. In our study, 90.6% (29/32) of patients in the RT group underwent intensity modulated RT (IMRT) rather than 2- or 3-dimensional conformal RT for whole breast irradiation. IMRT could improve the dose distribution of treatment fields in the breast; thus, it might result in superior breast cosmesis and less palpable induration [31]. Additionally, all patients in our study retained partial breast tissue and needed a small transfer flap rather than replacement of the whole breast tissue with a flap, which provided restoration with a natural texture, shape, and volume of the breast.

Although a few studies have reported data of aesthetic results after RT for patients who received IABR after BCS, aesthetic evaluations at different timepoints after RT and a comparative analysis between aesthetic results for patients with and without RT are still lacking. One early study [17] that included 34 patients who underwent RT at 4 to 6 weeks after IABR reported that the proportion of very good or good cosmetic outcomes was only 88.2%, which was lower than that of our study (96.9%). This difference might be due to the higher proportion of unaesthetic scars (8.9%) and marked fibrosis (2.9%) caused by the surgical methods used in that study. Unlike that study, we used LD flaps or FDFG to fill the defect through the primary incision so that an additional incision was avoided. Additionally, heterogeneity in the timepoints at which aesthetic outcomes were evaluated may be another reason. In our study, we evaluated cosmetic outcomes at different timepoints before and after radiotherapy and IABR. Additionally, the cosmetic outcomes of patients in the non-RT group were evaluated at the same timepoints. Thus, we observed that compared with the non-RT group, the addition of radiotherapy in the RT group did not affect cosmetic outcomes, indicating that RT might be a feasible option for breast cancer patients undergoing IABR after BCS because of durable cosmetic outcomes.

Postoperative flap volume changes are usually used to assess how much of the flap volume will ultimately remain. There is still a paucity of literature on flap volume changes following autologous flap breast reconstruction. Kimura et al. [32] described a maximum decrease in fat volume of 75.1% at 1 year after the operation, while Wilting et al. [33] showed a final overall flap volume decrease of 88.9% after 6 months. Rochlin et al. [27] found the incidence rate of flap volume loss events to be 16.9% among patients who received radiotherapy following IABR after mastectomy, but data on the impact of RT on flap volume changes are lacking. In this study, we assessed the changes of flap size in the three dimensions instead of volume changes, and our findings indicated that the size of the flap decreased slowly and tended to stabilize at the T1, T6, and T12 timepoints regardless of RT. Thus, RT had no adverse effect on flap size compared with the non-RT group. It is noteworthy that the flap size decreased obviously at an average of 4 months, which was shorter than what has been reported in previous studies [33, 34]. This may be related to the relatively smaller flap size required for BCS than for mastectomy, differences in patient populations or flap donor sites, baseline measurement times, measurement techniques used to evaluate flap size, and host conditions. Currently, there are no reports on the factors that affect the initial flap size after autologous reconstruction, but the reduction in flap size may be partially due to early postoperative factors such as apoptosis [35], postoperative edema, and inflammation. Flap denervation and ischemic changes caused by transient ischemia may also contribute to this reduction.

In our study, the common postoperative complications were partial necrosis of the flap, infection, hematoma, and seroma, the incidence rates of which were between 1.2 and 3.6%, which were lower than the 2.9 to 14.7% reported in previous studies [10, 17, 23, 36]. Different patient populations, excision extensions, flap sizes, and donor sites might lead to this difference. Patients from Western countries or undergoing mastectomy usually require larger flaps and more quantity of fat grafting to balance the esthetic and oncological aspects of the larger breast size, which can easily lead to impaired blood supply in the grafting site. In contrast, due to the small- to medium-sized breasts in the Asian population, all patients in our study underwent BCS followed by IABR with smaller flaps, which can avoid the local unsatisfactory blood supply. Additionally, most of the patients (57/84) in our study underwent IABR with LD flaps, so few patients experienced the risk of fat necrosis or liquefaction, which was commonly seen in the above studies. Nevertheless, there is still controversy regarding whether RT could increase the risk of postoperative complications [37]. By summarizing the data of 11 retrospective studies that included 316 patients who received RT following IABR after mastectomy, Rochlin et al. [27] concluded that postoperative complications of fat necrosis and contracture could be increased to 16.9 and 35.4%, respectively, by RT. However, our study showed that RT did not increase the risk of any postoperative complications like contracture or fat necrosis for patients who underwent IABR following BCS compared with the non-RT group. Additionally, no reconstructive complications occurred after RT in the median follow-up time of 33.3 months, although one patient had to terminate RT after receiving a dose of 36 Gy, due to partial flap necrosis. This is likely because the scope of surgical resection in our study was smaller than that of mastectomy, and retained some neurovascular function, which relatively reduced RT-related complications.

In this cohort study, patients who received IABR and RT did not experience any grade ≥ 3 RT-associated AEs. Of the eight patients (25%) with radiation pneumonitis, five (15.6%) and three (9.4%) cases experienced grade 1 and 2 radiation pneumonitis, respectively. Possible factors that contributed to the higher rate of grade 2 radiation pneumonitis than have been previously reported (0.8–3.7% in previous studies [38–40]) include higher ipsilateral lung dose exposure, systemic treatment with taxanes, and chronic inflammatory disease (chronic interstitial lung disease or asthma), due to their potential risk of increasing radiation-induced lung toxicity [40, 41]. In this study, the percent volume of ipsilateral lung receiving a dose ≥20 Gy (V20) and ≥ 5 Gy (V5) for the three patients who experienced grade 2 pneumonitis were 20–30% and 55–75%, respectively; all of them had previously received systemic treatment with taxanes, and two of them had chronic inflammatory disease (chronic interstitial lung disease or asthma). Notably, compared with the cases with no radiation dermatitis, among the patients who only received whole breast irradiation, 12 patients (37.5%) with boost irradiation to the tumor bed experienced grade 1 radiation dermatitis during RT, but the dermatitis gradually disappeared after radiotherapy. A previous report [42] showed that the tumor bed could be markedly replaced with flaps during reconstruction following BCS. Surgical bed clip placement in BCS is currently of the utmost importance in the definition of the RT boost volume to ensure precision, minimize geographical misses, and decrease normal tissue irradiation [43]. Although the identification of the tumor bed for local boost after oncoplastic breast surgery can be guided by intraoperatively placed titanium clips [44], this traditional location method may be prone to inaccuracy because of very large mammary gland translation, rotation, or excision. Therefore, a greater interaction between surgeons and radiation oncologists during boost planning has been viewed as a potential way to mitigate this complex process [45]. A recent study suggested that combining the clips with the redefinition of the flap on CT scan through a close cooperation between surgeons and radiation oncologists may provide more accurate tumor bed definition in patients undergoing partial breast reconstruction with chest wall perforator flaps [46]. However, it is an ideal ring-shaped boost that is difficult to reproduce in practice. Although tumor bed boosted radiotherapy following whole breast irradiation can reduce local recurrence rates, there is no evidence of a benefit for other oncological outcomes among BCS patients [47]. In light of this, it is necessary to conduct multidisciplinary discussions with breast surgeons, oncologists, and radiotherapists to accurately determine the location of the tumor bed and decide whether to perform tumor bed boosting during radiotherapy.

The introduction of oncoplastic techniques into clinical practice has the potential to reduce the risk of positive margins and ultimately the risk of local recurrence [48]. In our study, to ensure negative margins, we often performed a safer edge in the range of 1.5–3 cm, which is between the traditional BCS and mastectomy. RT was conducted in 38.1% of patients due to large tumors, lymph node metastases, or other high-risk factors. At a median follow-up of 33.3 months, all 84 analyzable patients remain alive, and median DFS time has not been reached, with a 2-year DFS rate of 97.6%. In total, 3.57% of patients had regional recurrences (3.13% in the RT group and 3.85% in the non-RT group). No patient experienced local recurrence in the subsequent 33.3 months of follow-up. This benefit for patients who underwent IABR may come from both RT and safer surgical margins.

However, there are also some limitations to this study. The inherent limitations of the study are its small sample size and retrospective nature. Additionally, the follow-up time is insufficient to definitively evaluate long-term outcomes. Some RT-associated late complications like fibrosis need further follow-up. Additionally, the optimal methods to assess cosmetic outcomes and flap size for these patients need to be further identified.

## Conclusion

This single-center retrospective cohort study showed that additional RT after IABR had no negative impact on aesthetic outcomes or flap size, and did not increase postoperative complications. Thus, adjuvant RT may be safely given to patients undergoing IABR following partial mastectomy. Further studies are needed to evaluate the long-term effects of RT on IABR.

## Supplementary Information

**Additional file 1: Table S1.** Cosmetic results of all patients

## Data Availability

The datasets used and/or analysed during the current study are available from the corresponding author on reasonable request.

## Abbreviations

IABR: Immediate autologous breast reconstruction
RT: Radiotherapy
BCS: Breast-conserving surgery
LD: Latissimus dorsi
FDFG: Free dermal fat graft
CT: Computed tomography
SD: Standard deviation
PR: Progesterone receptor
ER: Estrogen receptor
AEs: Adverse events
IMRT: Intensity modulated radiotherapy
