# Supplementary material for: Short-term outcomes and safety of radiotherapy for immediate breast reconstruction with autologous flap transfer following breast-conserving surgery
Source: BMC Cancer. 2021 Mar 2;21:214. doi: 10.1186/s12885-021-07915-4 (PMC7923603; doi:10.1186/s12885-021-07915-4)
Supplement: Supplementary file 1 — Additional file 1: Table S1. Cosmetic results of all patients [file 12885_2021_7915_MOESM1_ESM.docx]

Table S1. Cosmetic results of all patients

| Cosmetic results, n (%) | pre-RT | 1 month after RT | 6 months after RT |
| --- | --- | --- | --- |
| Scar |  |  |  |
| Very good | 19 (22.6) | 33 (39.3) | 44 (52.4) |
| Good | 65 (77.4) | 62 (60.7) | 40 (47.6) |
| Satisfactory | 0 | 0 | 0 |
| Poor | 0 | 0 | 0 |
| Shape |  |  |  |
| Very good | 20 (23.8) | 27 (32.1) | 45 (53.6) |
| Good | 64 (76.2) | 57 (67.9) | 39 (46.4) |
| Satisfactory | 0 | 0 | 0 |
| Poor | 0 | 0 | 0 |
| Symmetry |  |  |  |
| Very good | 24 (28.5) | 30 (35.7) | 43 (51.2) |
| Good | 60 (65.6) | 54 (64.3) | 41 (48.8) |
| Satisfactory | 0 | 0 | 0 |
| Poor | 0 | 0 | 0 |
| Skin color |  |  |  |
| Very good | 18 (21.4) | 16 (19.0) | 55 (65.5) |
| Good | 64 (76.2) | 54 (64.3) | 28 (33.3) |
| Satisfactory | 2 (2.4) | 14 (16.7) | 1 (1.2) |
| Poor | 0 | 0 | 0 |
| Nipple position | |  |  |
| Very good | 18 (25.0) | 27 (37.5) | 43 (62.5) |
| Good | 63 (68.8) | 55 (56.3) | 40 (34.4) |
| Satisfactory | 2 (3.1) | 2 (6.2) | 1 (3.1) |
| Poor | 1 (3.1) | 0 | 0 |
| Overall |  |  |  |
| Very good | 13 (15.5) | 11 (13.1) | 22 (26.2) |
| Good | 64 (76.2) | 59 (70.2) | 61 (72.6) |
| Satisfactory | 6 (7.1) | 13 (15.5) | 1 (1.2) |
| Poor | 1 (1.2) | 1 (1.2) | 0 |

Note: RT, radiotherapy.
